# Viscosity and Rheological Properties of Graphene Nanopowders Nanofluids

**DOI:** 10.3390/e23080979

**Published:** 2021-07-29

**Authors:** Abderrahim Bakak, Mohamed Lotfi, Rodolphe Heyd, Amine Ammar, Abdelaziz Koumina

**Affiliations:** 1 Laboratoire Interdisciplinaire de Recherche en Bioressources, Énergie et Matériaux (LIRBEM), ENS, Cadi Ayyad University, Marrakech 40000, Morocco; abderrahim.bakak@gmail.com (A.B.); koumina@uca.ac.ma (A.K.); 2 Materials, Energy & Environment Laboratory (LaMEE), FSSM, Cadi Ayyad University, Marrakech 40000, Morocco; lotfimohamed_1999@yahoo.fr; 3 Laboratoire Angevin de Mécanique, Procédés et innovAtion (LAMPA), Arts et Métiers ParisTech, Boulevard du Ronceray 2, BP 93525, CEDEX 01, F-49035 Angers, France; Amine.AMMAR@ensam.eu

**Keywords:** graphene nano-powder, thermal nanofluid, rheological behavior, Carreau nanofluid, lubrication effect, Vallejo law

## Abstract

The dynamic viscosity and rheological properties of two different non-aqueous graphene nano-plates-based nanofluids are experimentally investigated in this paper, focusing on the effects of solid volume fraction and shear rate. For each nanofluid, four solid volume fractions have been considered ranging from 0.1% to 1%. The rheological characterization of the suspensions was performed at 20 ${}^{\circ }$C, with shear rates ranging from $10^{-1}\;s^{-1}$ to $10^{3}\;s^{-1}$, using a cone-plate rheometer. The Carreau–Yasuda model has been successfully applied to fit most of the rheological measurements. Although it is very common to observe an increase of the viscosity with the solid volume fraction, we still found here that the addition of nanoparticles produces lubrication effects in some cases. Such a result could be very helpful in the domain of heat extraction applications. The dependence of dynamic viscosity with graphene volume fraction was analyzed using the model of Vallejo et al.

## 1. Introduction

Global warming and environmental disasters are current events that demonstrate the urgency of a better consideration of renewable energy sources. According to the International Energy Agency (IEA), during 2018, the fossil fuel share represented 81% of the 14,314 Mtoe of the world’s primary energy demand, while the share of renewable energy was only 9.7%. According to the IEA, improving energy efficiency is the central factor that will enable the world to move towards a sustainable development scenario. Unfortunately, the IEA also noted a clear slowdown in global progress on energy efficiency in its 2019 report [1], which is of serious concern in the objective to meet global climate targets and other sustainable energy goals. It is therefore vital to improve energy efficiency at all levels of fossil resource use and consequently every reliable contribution in this direction is welcome.

Heat transfer plays a vital role in many industrial and technical applications, ranging from cooling of heat engines or high-power transformers to heat exchangers used in hot water solar panels, refrigeration systems, or power plants. Unfortunately, usual heat transfer fluids (HTFs) such as water (Wa), thermal oils (TOs), ethylene-glycol (EG), or lubricating oils (LOs) all have thermal conductivity less than one unity ($k<1\;W\cdot m^{-1}\cdot K^{-1}$), and this is a significant obstacle in improving the efficiency in thermal energy transfer or extraction.

According to Fourier’s law $j_{Q}=-k\nabla T$, increasing the thermal conductivity *k* of HTFs will result in increasing the conductive heat flux between solids and HTFs. Thus, one way to improve heat extraction is to combine the flow properties of HTFs with the high thermal conductivity of some solid materials, such as metals (Cu, Ag, Fe, etc.), metal oxides (CuO, Cu${}_{2}$O, SiO${}_{2}$, TiO${}_{2}$, Al${}_{2}$O${}_{3}$, etc.), or different carbon-based materials (carbon black (CB), carbon nanotubes (CNT), and nanohorns (CNH) or graphene (GR)).

However, the use of suspensions with micrometer-sized solid materials (micro- composites) would lead to many prohibitive problems, such as abrasion, sedimentation, and high risk of clogging. Fortunately, advances in nanotechnology now make it possible to synthesize a wide variety of highly thermally conductive solid nanoparticles (NPs), which can be stably suspended in HTFs to form nano-composites (nanofluids and nanolubricants) and impart interesting thermal properties for heat extraction, without the disadvantages mentioned above.

Nanofluids [2] are colloidal suspensions composed of solid nanoparticles (NPs), having at least one dimension that is nanometric in size (<100 nm), stably suspended in a thermal liquid such as water, ethylene glycol, or thermal oils [3,4,5]. Lubricating oil-based suspensions are also sometimes called nanolubricants [6]. The amazing thermal properties of nanofluids have been the subject of intense investigations in recent years [3,4,7,8,9]. The potential applications of these nano-suspensions are multiple and promising in various fields, such as cooling power electronic components, industrial and domestic air conditioning and cooling, heat extraction, and transport. As mentioned previously, these suspensions could constitute, under certain mechanical conditions of use which will be discussed later, a promising outlet for nanosciences in the field of energy saving [9,10,11].

Due to the very large contact areas provided by porous media [12,13], their use in heat exchangers could also be an interesting way to improve heat transfer (in ducts and pipes, for example). It could therefore be possible to combine the two aspects (thermal nanofluids and porous media) to further intensify heat extraction [14], provided of course that the addition of nanoparticles does not significantly increase the base fluid viscosity.

Nanofluids also have a wide range of applications in many other fields than thermal transfers. We can mention, for example, the very promising field of nanomedicine, where nanocarriers are used to allow the delivery of therapeutic and/or imaging agents directly to tumor cells [15,16].

Different kinds of nano-particles (NPs) have been considered so far to produce nanofluids. They can be prepared from polymeric, metallic, organic, and inorganic materials, in the form of tubular (e.g., carbon nanotubes), spherical (metals and oxides) or layered (graphene) structures [10,17,18,19,20,21,22,23]. Among these various materials, graphene is a very promising candidate because of its exceptional physical properties, including: a high value of charge carrier mobility [20], exceptional transport performances [22], high specific surface area [24], high thermal conductivity [18], and a significant Young’s modulus [25]. These properties rank this allotropic variety of carbon in the category of the most suitable materials for the preparation of thermal nanofluids, which are sought after mainly to improve heat extraction capacities.

While the most cited carbon-based nanomaterials for nanofluids applications are carbon nanotubes [26,27,28,29,30,31], recently other structures (such as graphene nanoplatelets (GNPs) and reduced graphene oxide (RGO)) have become more widespread [32]. Several researchers have studied the rheological properties of different graphene based nanofluids [9,32,33,34,35,36]. Monireh et al. [35] examined the impact of several parameters on the rheological properties of glycerol and multilayer graphene nanofluids. Their results show that the viscosity increases with the raise in the solid mass fraction (between 0.0025 and 0.0200). They reported an increase in the viscosity (401.49%) of glycerol for 2% graphene nano-sheets fraction, at shear rates of 6.32 s${}^{-1}$ and at a temperature of 20 ${}^{\circ }$C. In addition, Kole et al. [34] examined and evaluated the effect of graphene nano-sheets, added to the base fluid (distilled water + ethylene glycol). Their results showed a non-Newtonian behavior with the appearance of a reduction in viscosity by shearing, and an increase of 100% compared to the basic fluid for a graphene volume fraction of 0.395%. In their paper, Kazemi et al. [36] examined the effect of adding Silica and Graphene nanoparticles (using volume fractions 0.05%, 0.1%, 0.25%, 0.5%, and 1%) on viscosity of water. Their experimental results revealed non-Newtonian pseudoplastic behavior of Graphene/water nanofluids. Ahammed et al. [37] have studied the effect of volume fraction (0.05–0.15%) and the temperature (10–90 ${}^{\circ }$C) on the viscosity of nanofluids containing graphene nanosheets dispersed in water. They found that the nanofluid water–graphene viscosity decreases with increasing temperatures and increases with the volume fraction of the nanosheets. An average increase of 47% in viscosity has been noted for 0.15% volume fraction of graphene at 50 ${}^{\circ }$C.

While, from a heat transfer point of view, thermal conductivity is an essential property of thermal nanofluids, from a practical point of view, the dynamic viscosity of these suspensions is an essential property for applications involving fluid flow, as heat transfer and mechanical efficiency are deeply impacted by the viscosity of the fluid [27,32,38]. The addition of nanoparticles to a base fluid can significantly alter its rheological properties, inducing, for example, non-Newtonian behaviors, and, moreover, it can lead to a significant increase of head losses. These pressure losses and rheological behavior alterations can represent a serious limitation to the industrial use of thermal nanofluids [32]. It is therefore important to study them systematically in order to predict the best operating ranges of the considered thermal nanofluids. This is thus the main motivation of the present research.

The improvement of the thermal conductivity and the modification of the viscosity of nanofluids strongly depend on several parameters, among which the size and concentration of the nanoparticles, their nature and shape, the nature of the base fluid, the operating temperature and the shear rate [32,33,39,40].

This paper presents an experimental study of the rheological properties of two thermal nanofluids based on an allotropic variety of graphene nanoparticles, called graphene nano-platelets (GNPs). In this study, the GNPs nanoparticles were dispersed in two kinds of base fluids, with quite different viscosities: an industrial lubricating oil (LO) and ethylene glycol (EG). We are concerned here with the study of the influence of solid particles concentration and of shear rate on the rheological behavior of the suspensions under investigation. Preparation and characterization of the suspensions used in the study are presented in the first part of the paper (Experimental Methods). Then, experimental results are presented and analyzed in terms of the influence of solid volume fraction and shear rate on the rheological properties of the two graphene-based suspensions (Results and Discussion). The experimental results are compared with different models (Carreau–Yasuda and Cross in regard to the shear rate and Vallejo and Maron–Pierce for the solid volume fraction). Conclusions and perspectives for future investigations are finally proposed (Conclusions and Perspectives).

## 2. Experimental Methods

### 2.1. Materials

Ethylene glycol (EG) (Sigma-Aldrich, BioUltra ≥ 99.5%) and a lubricating oil (LO) (Fuchs, ISO VG 68 RENEP CGLP) were used as base fluids. Table 1 shows the measured $\eta_{\mathrm{mea}}$ and reference $\eta_{\mathrm{ref}}$ viscosity values of pure base liquids at 20 ${}^{\circ }$C. The reference viscosity of EG is given by [41], while the reference value for LO is given by the manufacturer Fuchs. The uncertainty $\varepsilon_{\eta }$ between measured and reference values is also given in % in Table 1.

**Figure 1:**
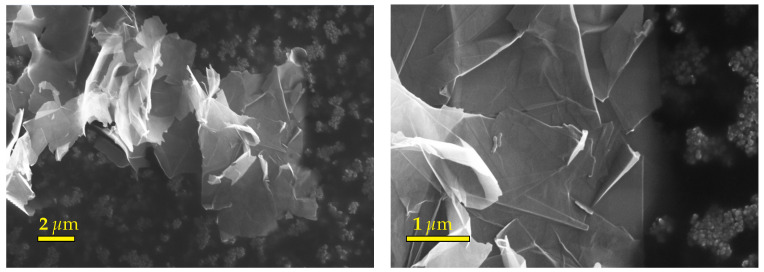
SEM images of GNP nano-sheets, made at a working distance (WD) of 9.4 mm, an electron high tension (EHT) of 15.00 kV and noise reduction by line average filtering.

The GNPs were purchased from the Graphene Supermarket company (GSc) and used as is. The dry powder of GNPs has a black color, a purity of 99.2%, and a density ρ=2.25 g·cm${}^{-3}$ (from GSc). Figure 1 shows the scanning electron microscope (SEM) images of the GNP sheets (performed at LAMPA with a Zeiss Supra 25 microscope, allowing a 1.5 nm resolution at 20.00 kV). These images clearly show the structural morphology of GNPs in the form of nano-sheets with an average thickness of 3 nm (according to GSc). These sheets are thus composed of 3 to 8 graphene mono-layers. Figure 1 also shows that the nano-sheets are aggregated and overlap randomly.

**Figure 2:**
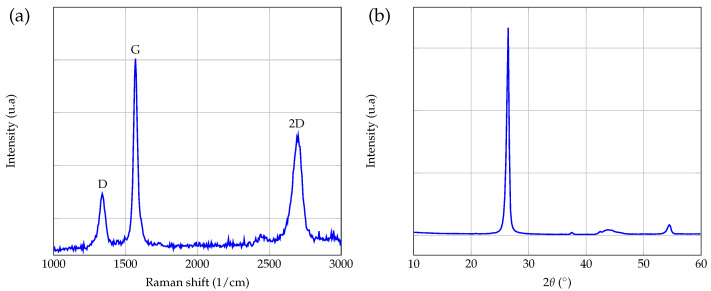
Raman spectrum (**a**) and XRD spectrum (**b**) of the GNPs powder used in this study.

The nano-powders were analyzed by Raman spectroscopy, a technique that is commonly used to characterize graphitic materials. Figure 2a shows a typical Raman spectrum obtained with the powders used in this study. The three main peaks characteristic of graphene-based materials are present, with usual relative intensities and widths: G (∼1580 cm${}^{-1}$) and 2D (∼2690 cm${}^{-1}$) peaks that are always present in the case of graphene and the D peak (∼1350 cm${}^{-1}$), which indicates the presence of defects [42]. Raman spectra were performed using a Confotec MR520 Raman spectrometer at λ=532 nm, with an analysis time of 30 s.

X-ray diffraction (XRD) analysis was also conducted on the nano-powders. Figure 2b shows that the diffraction pattern of graphene powders presents two main peaks at 2θ∼$27^{\circ }$ and 2θ∼$54^{\circ }$, which are very close to the diffraction peaks of graphite, as mentioned in [43].

### 2.2. Graphene Suspensions

Different masses of GNPs were dispersed in 20 mL of each base fluid, to obtain the following solid volume fractions: ϕ=0.1%, 0.25%, 0.5%, and 1%. No dispersing agent or surfactant has been used in the formulation. Each mixture has been stirred with a magnetic stirrer for 48 h, to ensure a uniform dispersion of the nano-particles in the base fluid. In order to limit the initial agglomeration of the nano-particles in the base fluid, the solutions were exposed to moderate sonication (LEO ultrasonic bath, oscillation frequency 46 kHz and power 80 W) for 2 h. The samples, contained in closed vials, were immersed in a water bath at room temperature. No significant variation of the samples temperature was observed during the sonication process. Next, all suspensions were stored at room temperature in hermetic containers. No observable phase separation has been detected before and after rheological measurements. In all the cases considered here, the preparation of suspensions with a solid volume fraction of 2% has led to samples that were no longer liquid but rather pasty and that behaved like gels (no flow under the effect of gravity, by turning the vial upside down). This study is therefore limited to solid volume fractions of less than 2%.

### 2.3. Suspensions Characterization

**Figure 3:**
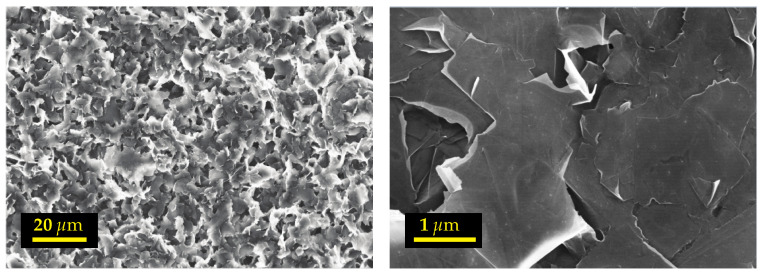
SEM characterization of EG-GNPs-0.25 nanofluid.

To study the state of dispersion of the nano-particles in the base fluid and to evaluate the presence and size of graphene aggregates [33,44,45,46,47], samples of each suspension were analyzed by SEM after drying (Figure 3 shows an example of nanofluid based on ethylene glycol, for a solid volume fraction ϕ=0.25%: EG-GNPs-0.25). One drop of each sample was collected, placed on the SEM grid and then slowly dried in an oven (see Table 2). Figure 3 shows that the graphene nano-sheets are uniformly dispersed, revealing no irreversible agglomerates, and that the morphology of these nano-sheets is not noticeably altered after the stirring and sonication steps.

The rheological study of the nanofluids was carried out with a rotational rheometer (Malvern Kinexus Pro), using a cone-plate geometry (1${}^{\circ }$–60 mm), temperature-controlled with a resolution of 0.01 ${}^{\circ }$C. The geometry and the liquid to be characterized were enclosed under a cover (hood), in order to improve the temperature homogeneity within the sample. All the dynamic viscosity measurements were performed with the same geometry. No particular experimental problems, such as material rejection or phase separation, were observed during the measurements.

### 2.4. Rheological Properties of Base Liquids

**Figure 4:**
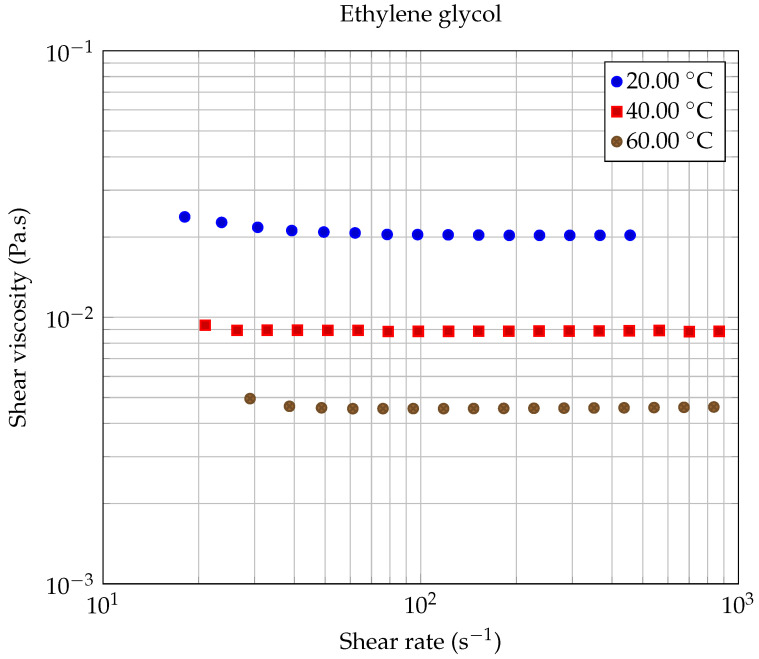
Rheological behavior of ethylene glycol at different working temperatures.

The first experiments were carried out on the base liquids, in order to evaluate the uncertainty of the rheometer. All measurements were repeated at least twice to check their reproducibility. Figure 4 shows the results of our measurements obtained for pure ethylene glycol, the less viscous base liquid used in the present study, at three working temperatures (20.00 ${}^{\circ }$C, 40.00 ${}^{\circ }$C, and 60.00 ${}^{\circ }$C). The values that we have measured for these three temperatures are quite close to those obtained by Chen et al. [48] and Sawicka et al. [41]. As expected, the dynamic viscosity (η) of ethylene glycol is independent of shear rate. Similar results were obtained for the lubricant oil. The base fluids used in this study behave like Newtonian fluids over the whole temperature range under investigation. The relative measurement uncertainty has been estimated to be on the order of 2% at 20.00 ${}^{\circ }$C.

**Figure 5:**
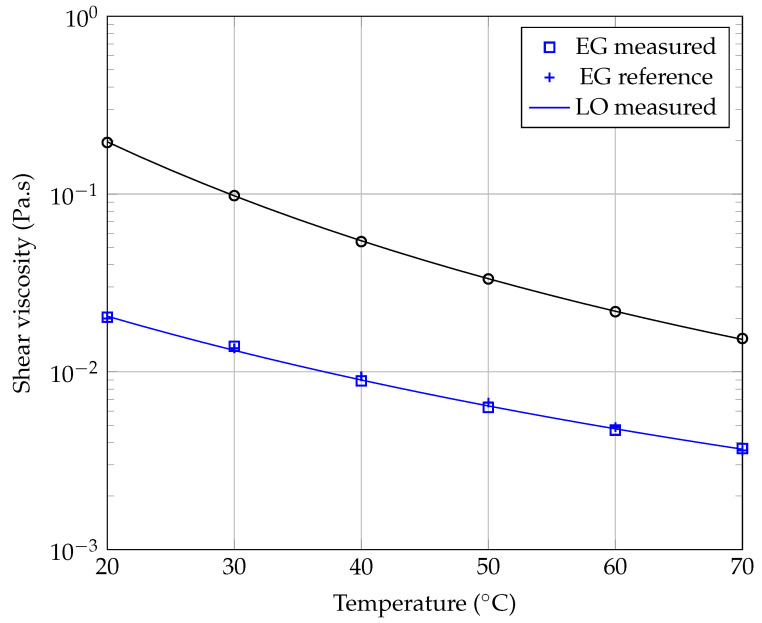
Evolution of the dynamic viscosity of base liquids as a function of temperature. The continuous lines represent model (1) with the respective coefficients of Table 3.

Figure 5 shows the evolution of dynamic viscosity as a function of temperature for each of the two base fluids used in our study. As expected, it is observed that the dynamic viscosity $\eta_{\mathrm{bf}}$ of these base fluids is a decreasing function of temperature, according [48] to usual laws of type:

(1) $$\ln\eta_{\mathrm{bf}}=A+1000\times \frac{B}{T+C},$$

where *T* is the absolute temperature, $\eta_{\mathrm{bf}}$ is the base fluid viscosity (in mPa·s), and *A*, *B*, and *C* are fluid specific constants. Even more specific laws can be used (see, for example, Bird et al. [49], chapter 1). It is also possible to write (1) in the so-called Vogel–Fulcher–Tamman form [50,51]:

(2) $$\eta_{\mathrm{bf}}=\eta_{0}e^{\frac{DT_{0}}{T-T_{0}}},$$

where $\eta_{0}$ (in Pa·s), $T_{0}$ (in K) and *D* are fitting parameters related to *A*, *B*, and *C* by the following relations: $\eta_{0}=10^{-3}\cdot e^{A}$, $T_{0}=-C$ and $D=-10^{3}\cdot B/C$.

Table 3 gathers the values of coefficients *A*, *B*, *C* and of the determination coefficient $R^{2}$ calculated for the two base fluids used in this study.

In [41], Sawicka et al. used an Arrhenius-type law to model their measurements of EG viscosity as a function of absolute temperature *T*:

(3) $$\eta_{\mathrm{EG}}=A\;\exp\left(\frac{B}{T}\right),$$

where $A=1.6\times 10^{-7}$ Pa·s and B=3440 K. Note that, using model (3), our experimental measurements led to the following values in the case of EG: $A=1.11\times 10^{-7}$ Pa·s and B=3548 K, with a coefficient of determination $R^{2}=0.9971$. These results confirm the consistency of the present viscosity measurements with those of Sawicka et al. Table 4 collects our measurements $\eta_{\mathrm{mea}}$ of viscosity versus temperature for ethylene glycol and compares them with the results $\eta_{\mathrm{ref}}$ obtained by Sawicka et al. in [41]. The corresponding uncertainties $\varepsilon_{\eta }$ are also given in %.

## 3. Results and Discussion

### 3.1. Rheological Behavior

**Figure 6:**
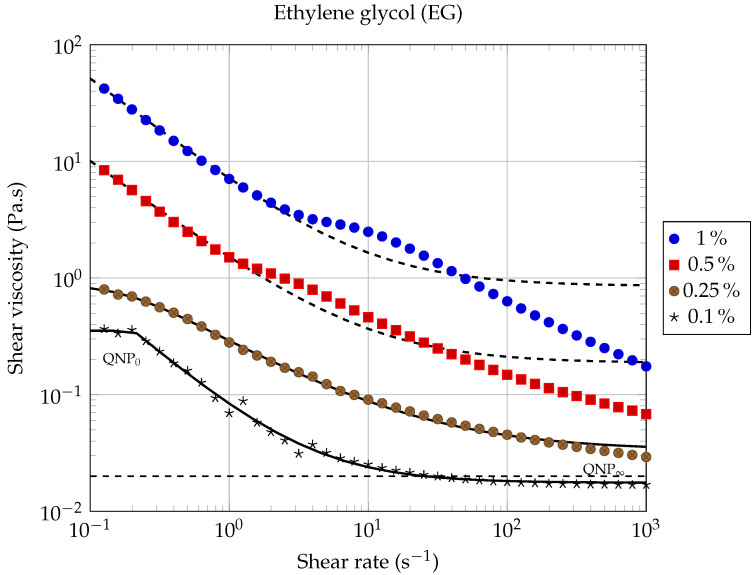
Dynamic viscosity of EG-GNP nanofluids as a function of shear rate, for different solid volume fractions, at 20.00 ${}^{\circ }$C. Solid and dashed lines correspond to the model (6), using the coefficients gathered in Table 5. The horizontal dashed line indicates the viscosity value of the base fluid.

**Figure 7:**
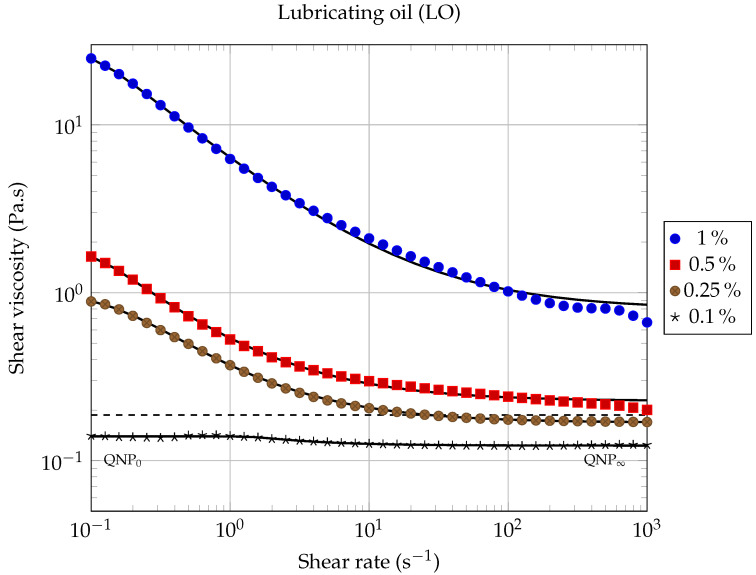
Dynamic viscosity of LO-GNP nanofluids as a function of shear rate, for different solid volume fractions, at 20.00 ${}^{\circ }$C. Solid lines correspond to the model (6), using the coefficients gathered in Table 5. The dashed line indicates the viscosity value of the base fluid.

Figure 6 and Figure 7 show the rheological behavior of the two nanofluids for different volume fractions, at a working temperature of 20.00 ${}^{\circ }$C, and as a function of the shear rate. Within the shear rates range investigated, it is observed that the rheological behavior of the nanofluid is strongly dependent on the solid volume fraction. For each of the two studied nanofluids, shear-thinning has been observed, which is more pronounced for higher solid volume fractions. As indicated in the literature [35], the decrease in viscosity as a function of shear rate could be attributed to a de-agglomeration effect of the graphene nanosheets or to the alignment of the nanosheets in the plane of flow during shearing [48], resulting in less viscous dissipation and consequently in a decreasing of the apparent viscosity of the suspension. As the volume fraction of GNPs suspended in the base fluid increases, shear-thinning deviation from Newtonian behavior becomes more and more pronounced. Very similar behaviors were observed by Vallejo et al. [51] using nanofluids composed of an ethylene-glycol:water mixture (50:50 vol%) and different carbon-based nanomaterials (carbon black, nanodiamonds, graphite/diamond mixtures and sulfonicacid-functionalized graphene nanoplatelets).

For each of the two types of nanofluids studied here, two quasi-Newtonian plateaus can be observed for the lowest solid volume fraction (ϕ=0.1%). The first one, denoted QNP${}_{0}$, is observed at low shear rates (see Figure 6 and Figure 7), while the second one, denoted QNP${}_{\infty }$, is observed at high shear rates. The QNP${}_{\infty }$ plateau is also present for the volume fraction (ϕ=0.25%), but only in the case of LO based nanofluids. It can be noted that the extent of each of these plateaus depends on both the nature of the base liquid considered and on solid volume fractions. The presence of such Newtonian plateaus in the rheological behavior of nanofluids based on carbonaceous nanomaterials has also been reported by Vallejo et al. [51].

**Figure 8:**
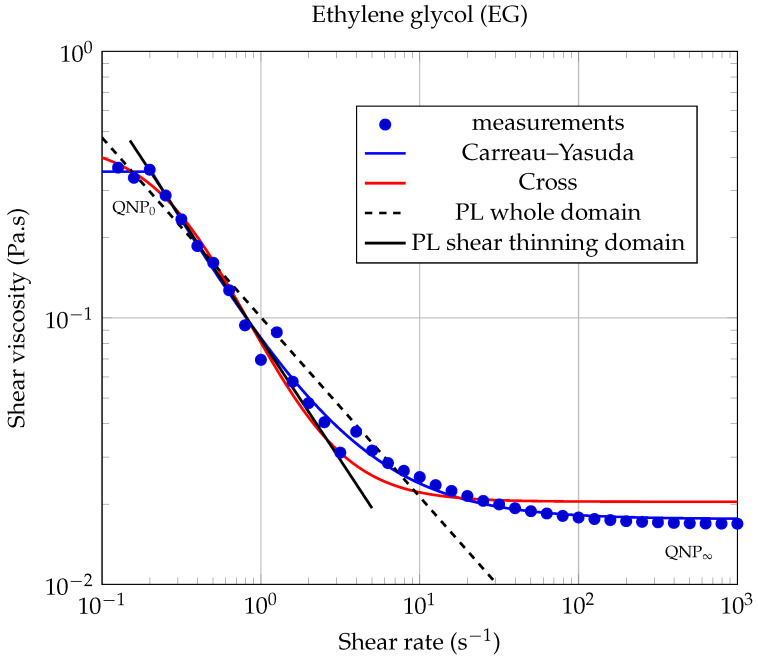
Dynamic viscosity of EG-GNP nanofluid as a function of shear rate, for the lowest solid volume fraction ϕ=0.1%, at $20.00{\;}^{\circ }$C. The Carreau–Yasuda model (6) has been plotted using the coefficients gathered in Table 5. In the shear thinning domain, PL modeling led to a quite good coefficient of determination $R_{\mathrm{PL}}^{2}=0.9981$.

**Figure 9:**
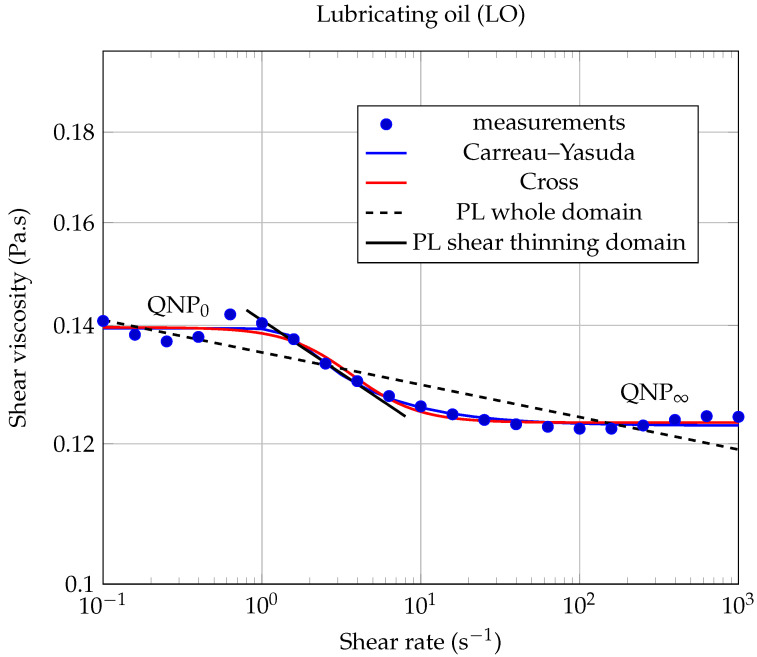
Dynamic viscosity of LO-GNP nanofluid as a function of shear rate, for the lowest solid volume fraction ϕ=0.1%, at $20.00{\;}^{\circ }C$. The Carreau–Yasuda model (6) has been plotted using the coefficients gathered in Table 5. In the shear thinning domain, PL modeling led to a coefficient of determination $R_{\mathrm{PL}}^{2}=0.9891$.

In the absence of Newtonian plateaus, the shear-thinning behavior of suspensions is often well described using a power law ([52] chapter 5, page 90), also known as the Ostwald–de Waele law (PL):

(4) $$\eta =k|\dot{\gamma }{|}^{n-1},$$

where $\dot{\gamma }$ is the shear rate, k>0 is the flow consistency index, and *n* is the flow behavior index (n<1 for shear-thinning behavior). From the results shown in Figure 6 and Figure 7, it is clear that a power law of type (4) cannot describe the whole contour shape of the flow curves for the different nanofluids studied here. It can only describe a small range of shear rates, corresponding to the shear thinning region. We have illustrated the inability of the power law (PL) to adequately describe the whole rheological behavior of our nanofluids, firstly in Figure 8 and Figure 9, where we have compared different rheological models in the case of the lowest solid volume fraction ϕ=0.1% and secondly, in Table 5, where the corresponding coefficient of determination $R_{\mathrm{PL}}^{2}$ has been calculated when applying the power law (4) over the whole shear rates domain.

In [51], Vallejo et al. used the Cross model (CM) to fit their rheological measurements:

(5) $$\eta =\eta_{\infty }+\frac{\eta_{0}-\eta_{\infty }}{1+{\left(k\cdot \dot{\gamma }\right)}^{m}},$$

where *m* and *k* are called the rate constant and the time constant, respectively, while $\eta_{0}$ and $\eta_{\infty }$ are the asymptotic values of dynamic viscosity corresponding to QNP${}_{0}$ and QNP${}_{\infty }$, respectively [51]. This law has shown to be more suitable than the power law to describe our measurements over the entire range of shear rates studied (see Figure 8 and Figure 9).

Our experimental data were also fitted using the Carreau–Yasuda (CY) model for shear-thinning fluids [53,54]:

(6) $$\frac{\eta -\eta_{\infty }}{\eta_{0}-\eta_{\infty }}={\left[1+{\left(\lambda \dot{\gamma }\right)}^{a}\right]}^{\frac{n-1}{a}},$$

where $\eta_{0}$ is the zero shear rate dynamic viscosity (corresponding to QNP${}_{0}$); $\eta_{\infty }$ is the high share rate dynamic viscosity (corresponding to QNP${}_{\infty }$) and, according to Kowalska et al., λ is a relaxation time characteristic of the studied fluid and *a* is a parameter characteristic of the transition width between the zero shear rate viscosity domain and the shear thinning domain. The values of the Carreau–Yasuda parameters, obtained by fitting our experimental results, are gathered in Table 5 and were used to plot the continuous and dashed lines (except horizontal lines) in Figure 6 and Figure 7. It can be seen from these figures that, at the lowest solid volume fractions used here (ϕ≤0.25%), the rheological behavior of each of the two types of nanofluids is well described by the CY model, over the whole range of shear rates investigated here.

It should be noted that the CY model consistently gave better results than the Cross model, for each of the nanofluids studied in this work (see the coefficients of determination $R_{\mathrm{CY}}^{2}$ and $R_{\mathrm{CM}}^{2}$ collected in Table 5). Therefore, we will discuss hereafter only the results given by the CY model.

It can be noticed that the CY model still applies here remarkably well for the highest solid concentrations studied in this work (ϕ=0.5% and ϕ=1.0%), but only at low shear rates.

This remark is particularly true in the case of ethylene glycol-based nanofluids (EG-GNPs), where it is observed for shear rates above 2 $s^{-1}$ that the rheological behavior completely fails the Carreau–Yasuda model (see Figure 6, dashed lines). These large deviations from the CY model certainly reflect the increasing influence of graphene–graphene and graphene–ethylene glycol interactions on the rheological properties of the suspension, as the solid volume fraction and the shear rate increase. This very particular rheological behavior has also been observed in some cases by Vallejo et al., for high solid mass fractions (see [51], Figure 3: 0.50wt%Nd97 and Figure 4e: 2.0wt%nD87 and nD97).

On the other hand, we have found that the CY model is particularly well suited for lubricating oil-based nanofluids (LO-GNPs) over the entire shear rates range, as can be seen from the curves plotted in Figure 7 and from the results gathered in Table 5. The value of the coefficient of determination $R_{\mathrm{CY}}^{2}$ is in this case very close to one, for three of the four LO-based nanofluids studied here.

The significant differences in rheological behaviors observed in this work clearly highlight the influence played by base liquid/GNPs interactions on the rheological properties of graphene nanopowder-based suspensions.

### 3.2. Effect of Solid Volume Fraction on Dynamic Viscosity

We now analyze the influence of suspending various graphene nanosheets volume fractions (ϕ=0.1%, 0.25%, 0.5% and 1.0%) on the dynamic viscosity of each of the two base fluids under investigation, at the working temperature T=293.15 K.

The dependence of the room temperature dynamic viscosity with solid volume fraction ϕ of EG-GNPs and LO-GNPs nanofluids has been compared to several widely used models, namely Einstein [55], Brinkman [56], Batchelor [57], or Krieger–Dougherty [58] laws (see Table 6). These laws, valid only for spherical nanoparticles, proved to be totally inadequate with the nanofluids studied here, which contain graphene nanosheets.

**Figure 10:**
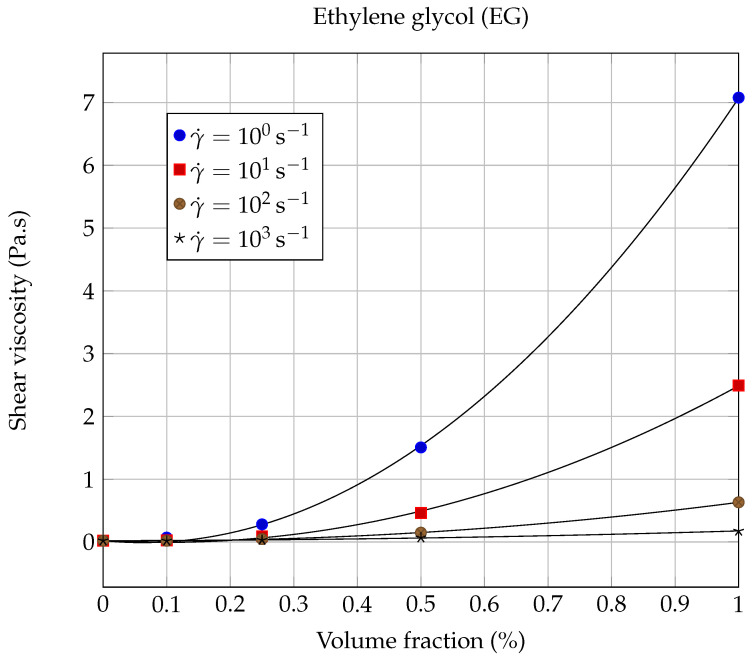
Evolution of the shear viscosity η of EG based nanofluids as a function of the solid volume fraction ϕ, at $20.00{\;}^{\circ }$C and different shear rates. The continuous lines correspond to the Vallejo model (8), with the coefficients collected in Table 7.

**Figure 11:**
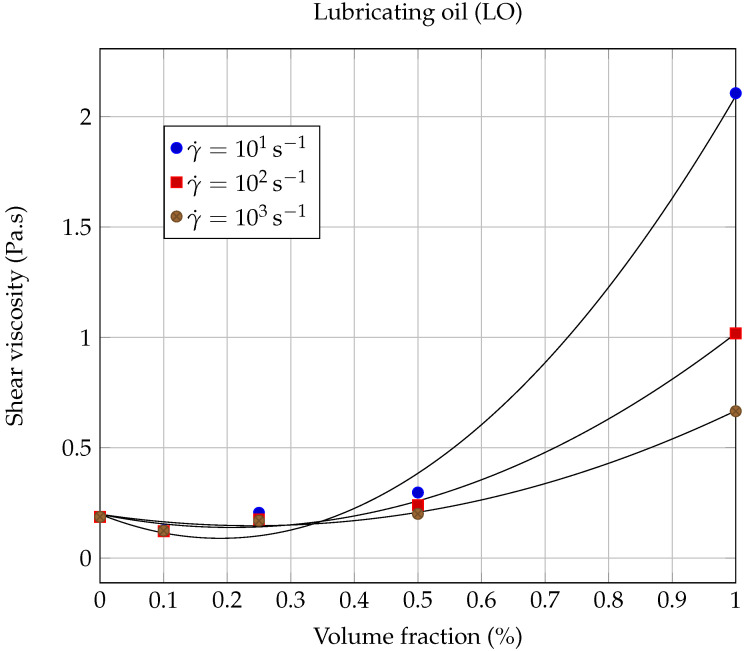
Evolution of the shear viscosity η of LO based nanofluids as a function of the solid volume fraction ϕ, at $20{\;}^{\circ }$C for moderate to high shear rates. The continuous lines correspond to the Vallejo model (8), with the coefficients collected in Table 8.

**Figure 12:**
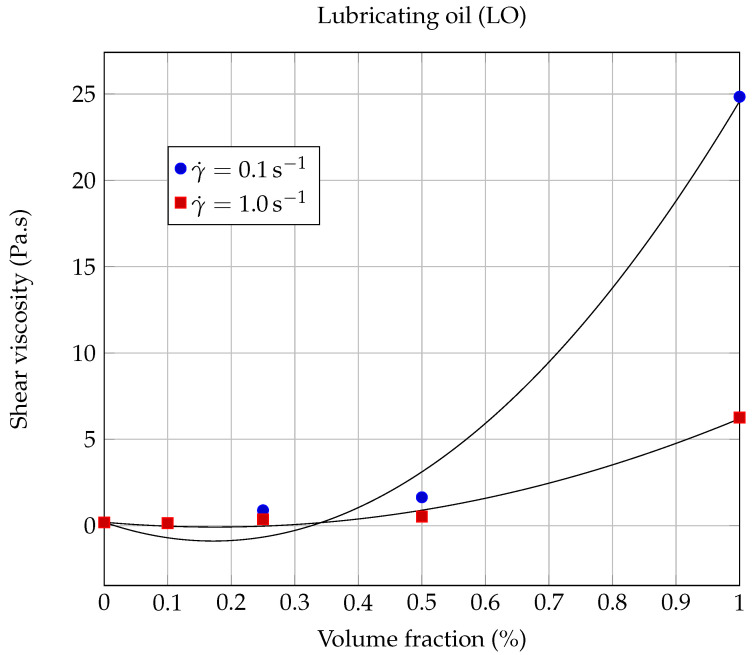
Evolution of the shear viscosity η of LO based nanofluids as a function of the solid volume fraction ϕ, at $20.00{\;}^{\circ }$C and low shear rates $\dot{\gamma }=0.1\;s^{-1}$ and $\dot{\gamma }=1.0\;s^{-1}$.The continuous lines correspond to the Vallejo model (8), with the coefficients collected in Table 8.

The dynamic viscosity η was calculated at $20.00{\;}^{\circ }C$ as a function of the solid volume fraction ϕ for each nanofluid, using the experimental results shown in Figure 6 and Figure 7, for the following shear rates: $\dot{\gamma }=0.1,\;1.0,\;10,\;100$ and $1000\;s^{-1}$. The evolution of dynamic viscosity as a function of the GNP volume fraction is shown in Figure 10 for the case of ethylene glycol-based nanofluids and in Figure 11 and Figure 12 in the case of lubricating oil.

The dynamic viscosity values of most of the present nanofluids were well modeled using the law proposed by Vallejo et al. [32,59]:

(7) $$\eta =\eta_{0}e^{\frac{DT_{0}}{T-T_{0}}}+Ee^{\frac{F}{T}}\cdot \phi -G\cdot \phi^{2}$$

where the parameters $\eta_{0}$, *D*, and $T_{0}$, whose values are gathered in Table 3, are specific to the base fluid; *E*, *F*, and *G* are fitting parameters. Since only one temperature *T* is considered in the present study, we rewrite Vallejo’s law in the following simplified form:

(8) $$\eta =\eta_{0}e^{\frac{DT_{0}}{T-T_{0}}}+E^{\prime }\cdot \phi -G\cdot \phi^{2}$$

The Maron and Pierce equation [32] was also used to model our measurements:

(9) $$\eta =\eta_{\mathrm{bf}}{\left(1-\frac{\phi }{\phi_{m}}\right)}^{-2}$$

where $\phi_{m}$ can be considered as a fitting parameter. This model, which gave good results for aqueous nanofluids containing graphene oxides [60], however, did not provide satisfactory results with the present nanofluids. Therefore, it will not be developed in the rest of this work.

More specifically, in the case of ethylene glycol a quasi systematic increase in the dynamic viscosity with the GNP volume fraction has been observed, whatever the shear rate considered (see Figure 6 and Figure 10); these results are similar to those already published in several studies [34,35,40,51,61,62,63,64]. However, we noticed a weak lubricating effect, at the limit of the measurement uncertainty, for the lowest volume fraction considered (ϕ=0.1%) and for shear rates above $40\;s^{-1}$.

Thus, it can be seen that from the point of view of mechanical performance that it is not very favorable to load ethylene glycol with graphene nanopowders, since it can significantly increase the effective dynamic viscosity of the suspension. This increase is more important as the shear rates considered are low. For example, for $\dot{\gamma }=1.0\;s^{-1}$ and ϕ=1%, we found a relative increase in viscosity that is equal to $\eta_{r}\left(1\;s^{-1}\right)=\eta /\eta_{\mathrm{bf}}\approx 350$, which is considerable. Relative increases of this order of magnitude, or even greater, have already been observed in the past with aqueous nanofluids based on carbon nanotubes (CNTs). For example, Ding et al. [65] observed huge variations in dynamic viscosity as a function of shear rate with water-CNT nanofluids. In the case of 0.5 wt.% CNTs suspended in water, they found at $25{\;}^{\circ }$C that $\eta_{r}\left(10^{3}\;s^{-1}\right)\approx 10$ while $\eta_{r}\left(1\;s^{-1}\right)\approx 10^{5}$.

The variations of the dynamic viscosity η of the suspensions as a function of the solid volume fraction ϕ were modeled using Vallejo’s law (8), at different shear rates $\dot{\gamma }$. As can be seen from the solid line curves shown in Figure 10 and from the values of the coefficients of determination collected in Table 7, it can be noticed that the model of Vallejo et al. applies quite well to the description of the evolution of EG based nanofluids viscosity as a function of graphene volume fraction ϕ, whatever the shear rate considered. These remarkable results confirm the interest of Vallejo’s model for ethylene glycol-based nanofluids containing graphene nanopowders.

Next, the viscosity values of the nanofluids based on lubricating oil and graphene nanopowders (LO-GNPs) are analyzed. Figure 7 shows a very interesting and promising rheological behavior, since the addition of GNPs leads here, for the lowest volume fraction ϕ=0.1%, to a decrease of the viscosity compared to the base fluid ($\eta_{r}\left(0.1\;s^{-1}\right)=\eta /\eta_{\mathrm{bf}}=0.75$ and $\eta_{r}\left(1000\;s^{-1}\right)=0.66$), rather than an increase, and this whatever the shear rate considered. Chen et al. [29] have also observed, in the case of a nanofluid prepared with a volume fraction of 0.4% carbon nanotubes (CNT) suspended in EG, that the effective viscosity of the suspension is lower than that of the base fluid, due to a lubrication effect of nanoparticles, which can also be assumed here to be the reason for the viscosity decrease. For higher volume fractions given by 0.5% and 1%, the viscosity of the nanofluid becomes greater than that of the base fluid, whatever the shear rate considered. These observations reflect a non-monotonic behavior of the dynamic viscosity as a function of the solid volume fraction that can be seen in Figure 11. The possibility of a lubrication effect, in the case of the LO-GNP nanofluids for solid volume fractions, which should allow a significant improvement in heat extraction, is a very encouraging result for the use of these liquids from an industrial point of view.

As can be seen from Figure 11 and Table 8, Vallejo’s model gave adequate results for moderate ($\dot{\gamma }=10^{1}\;s^{-1}$; $R^{2}=0.9935$) to high shear rates ($\dot{\gamma }=10^{2}\;s^{-1}$; $R^{2}=0.9954$ and $\dot{\gamma }=10^{3}\;s^{-1}$; $R^{2}=0.9900$). On the other hand, the agreement is much less beneficial (see Figure 12) for the two lower shear rates considered here: $\dot{\gamma }=0.1\;s^{-1}$ ($R^{2}=0.9886$) and $\dot{\gamma }=1.0\;s^{-1}$ ($R^{2}=0.9884$).

In contrast to the case of EG-GNP nanofluids, it can be deduced from the present measurements that the viscosity of LO-GNP nanofluids seems not to verify Vallejo’s law correctly at low shear rates. Since the nanoparticles are of the same nature for both types of nanofluids considered in this study, this difference in behavior should probably be attributed to the fluid-GNP interactions.

## 4. Conclusions and Perspectives

An experimental study of the rheological properties of two different graphene based nanofluids was presented for the following base fluids: ethylene glycol and an industrial lubricating oil. The influence of nanoparticles concentration on the rheological properties of the suspensions has been systematically studied, for different solid volume fractions (0.1%, 0.25%, 0.5% and 1%) at a working temperature of $20.00{\;}^{\circ }$C. The rheological properties of the suspensions were analyzed using both the Carreau–Yasuda and Cross models for shear thinning liquids. For each of the two types of nanofluids considered, the Carreau–Yasuda model gave the best results, the agreement being particularly good at low graphene concentrations (ϕ≤0.25%). However, the presence of graphene at higher concentrations can lead to deviations from the Carreau–Yasuda law, which become more significant at high shear rates.

The Vallejo model was successfully applied to ethylene glycol-based nanofluids whatever the shear rate considered. In the case of lubricating oil-based nanofluids, the dependence of the viscosity on the solid volume fraction is moderately well described by Vallejo’s law for low shear rates. Further research will be needed to determine whether other base liquids also escape Vallejo’s law for low shear rates in the case of graphene-based nanofluids.

The suspensions studied in this work have exhibited a wide variety of original rheological behaviors. A lubrication effect has been demonstrated for the nanofluid based on lubricating oil, for which the viscosity decreases with the addition of graphene nano-sheets at ϕ=0.1%. This interesting behavior allows us to consider industrial applications for this nanofluid in the field of heat extraction, for example, without sacrificing the mechanical performance.

Future work will focus on the rheological behavior of these two types of nanofluids as a function of temperature, but also on the thermal and thermodynamic properties (thermal conductivity, specific heat, solidification temperature) and on the electrical and dielectric properties (electrical conductivity and dielectric permeability).

## Figures and Tables

**Table 1:** Measured $\eta_{\mathrm{mea}}$, reference $\eta_{\mathrm{ref}}$ viscosity values (in mPa·s) and uncertainty $\varepsilon_{\eta }$ (in %) of pure base liquids at 20 ${}^{\circ }$C.

Base Liquid	$\eta_{\mathrm{ref}}$	$\eta_{\mathrm{mea}}$	$\varepsilon_{\eta }$
Ethylene Glycol (EG)	19.9	20.3	2.0
Lubricating Oil (LO)	195	187	4.3

**Table 2:** Characteristics of oven sample processing.

Base Fluid	EG	LO
Oven duration	12 h	72 h
Temperature	100 ${}^{\circ }$C	220 ${}^{\circ }$C

**Table 3:** *A*, *B*, and *C* coefficients of Equation (1) and $\eta_{0}$, $T_{0}$, and *D* coefficients of Equation (2), calculated for the two base fluids used in this study.

Liquids	*A*	*B*	*C*	$\eta_{0}/10^{-5}$	$T_{0}$	*D*	$R^{2}$
EG	−3.202	0.813	−162.5	40.68	162.5	5.003	0.9974
LO	−2.353	0.757	−194.0	95.08	194.0	3.902	0.9998

**Table 4:** Ethylene Glycol viscosity values (in Pa·s) as a function of temperature.

*T* (${}^{\circ }$C)	20.00	30.00	40.00	50.00	60.00	70.00	80.00
$\eta_{\mathrm{mea}}$ **(Pa·s)**	0.0203	0.0139	0.0089	0.0063	0.0047	0.0037	0.0029
$\eta_{\mathrm{ref}}$ **(Pa·s)**	0.0199	0.0135	0.0094	0.0067	0.0049	0.0036	0.0027
$|\varepsilon_{\eta }|$ **(%)**	2.0	2.8	5.3	6.0	4.1	2.0	5.8

**Table 5:** Values of the Carreau–Yasuda parameters $\eta_{0}$, $\eta_{\infty }$, *a*, λ, and *n* obtained by fitting the experimental results. BF means base fluid (EG: Ethylene Glycol and LO: Lubricating Oil) at (@) solid volume fraction ϕ (in %). $R_{\mathrm{CY}}^{2}$ and $R_{\mathrm{CM}}^{2}$ are the determination coefficients corresponding to the Carreau–Yasuda and the Cross models, respectively. $R_{\mathrm{PL}}^{2}$ is the determination coefficient of the power law model, which has been applied only for ϕ=0.1%.

BF@ϕ	$\eta_{0}$ (Pa·s)	$\eta_{\infty }$ (Pa·s)	*a*	λ	*n*	$R_{\mathrm{CY}}^{2}$	$R_{\mathrm{CM}}^{2}$	$R_{\mathrm{PL}}^{2}$
EG@0.1	0.3535	$1.7574\times 10^{-2}$	123.84	4.8878	−0.0216	0.9967	0.9910	0.9689
EG@0.25	0.8731	$3.3330\times 10^{-2}$	2.3583	5.7222	0.3245	0.9992	0.9984	–
EG@0.5	19.776	0.1871	21.873	21.731	0.1295	0.9969	0.9969	–
EG@1.0	117.11	0.8536	18.701	25.214	0.0981	0.9978	0.9978	–
LO@0.1	0.1395	0.1229	7.3295	0.6650	0.0861	0.9753	0.9696	0.8217
LO@0.25	1.0120	0.1683	1.9802	7.1384	0.2703	0.9999	0.9996	–
LO@0.5	1.7795	0.2264	4.8753	9.7821	0.2927	0.9995	0.9989	–
LO@1.0	27.642	0.8118	4.1630	9.9430	0.3159	0.9998	0.9992	–

**Table 6:** Some models commonly used to estimate the viscosity of micro-dispersions as a function of the solid particles volume fraction. The intrinsic viscosity [η] has a typical value of 2.5 for spherical particles, $\phi_{m}$ is the maximum particle packing fraction (which has been chosen here as an adjustment parameter) and usually 5.2≤α≤7.6.

Models	Einstein	Brinkman	Batchelor	Krieger–Dougherty
$\eta /\eta_{\mathrm{bf}}=$	1+2.5ϕ	$1/{\left(1-\phi \right)}^{2.5}$	$1+2.5\phi +\alpha \phi^{2}$	${\left(1-\frac{\phi }{\phi_{m}}\right)}^{-\left[\eta \right]\phi_{m}}$

**Table 7:** Values of the Vallejo parameters $E^{\prime }$ and G obtained by fitting the experimental results of EG based nanofluids, for different shear rates, at $20.00{\;}^{\circ }C$.

Shear Rate $\left(s^{-1}\right)$	$E^{\prime }\;(\mathrm{Pa}\cdot s)$	G(Pa·s)	$R^{2}$
0.1	−8.3824	−50.4425	0.9996
$10^{0}$	−0.9938	−8.0442	0.9998
$10^{1}$	−0.5826	−3.0493	0.9994
$10^{2}$	−0.0908	−0.7015	0.9999
$10^{3}$	0.0143	−0.1411	0.9953

**Table 8:** Values of the Vallejo parameters $E^{\prime }$ and G obtained by fitting the experimental results of LO based nanofluids, for different shear rates, at $20.00{\;}^{\circ }C$.

Shear Rate $\left(s^{-1}\right)$	$E^{\prime }\;(\mathrm{Pa}\cdot s)$	G(Pa·s)	$R^{2}$
0.1	−12.723	−37.105	0.9886
$10^{0}$	−3.2195	−9.2146	0.9884
$10^{1}$	−1.1533	−3.0482	0.9935
$10^{2}$	−0.5789	−1.3981	0.9954
$10^{3}$	−0.4336	−0.9024	0.9900
